# Chemotherapy in a Patient With G6PD Deficiency and Advanced Testicular Cancer

**DOI:** 10.1200/JGO.17.00034

**Published:** 2017-07-17

**Authors:** Deise Uema, Denyei Nakazato, Cheng Tzu Yen, Eduardo Perrone, Diogo Assed Bastos, Gilberto de Castro

**Affiliations:** **Deise Uema**, **Cheng Tzu Yen**, **Diogo Assed Bastos**, and **Gilberto de Castro Jr**, Hospital Sírio-Libanês; **Denyei Nakazato**, Santa Paula Hospital; and **Eduardo Perrone**, Federal University of São Paulo, São Paulo, Brazil

## INTRODUCTION

G6PD is the most common enzymatic deficiency in humans,^1,2^ affecting approximately 5% of the world's population.^3^ Currently, there are more than 180 reported genetic variants of G6PD and its expression can vary from a mild (class V) to a severe deficiency of the enzyme (class I).^3^ G6PD deficient erythrocytes have difficulties in handling oxidative stress and, subsequently, are more susceptible to lysis.^3^ Antimalarials (dapsone, primaquine, methylene blue) are the classic therapeutic agents associated with acute hemolytic anemia but several other drugs are deemed as possible causes of hemolysis in G6PD deficient patients.^2,4,5^ Until the present moment, little is known about the prevalence of G6PD deficiency in cancer patients, and data regarding the use and safety of chemotherapy treatments in this population in the literature is extremely scarce.^6^ Here we describe the case of a young man with advanced testicular germ cell tumor treated with cisplatin-based chemotherapy (bleomycin, etoposide and cisplatin).

## CASE REPORT

A 26-year-old man with known G6PD deficiency presented at the hospital in November 2016 complaining of right testicle enlargement for the past 2 months without other significant symptoms. A scrotal ultrasound was performed and showed a testicle of increased size (27.3 cm^3^) with diffuse heterogeneity. A computed tomography scan of the chest, abdomen, and pelvis revealed multiple lung nodules up to 28 mm and thoracic and retroperitoneal lymph nodes suggestive of advanced germ cell tumor. Serum tumor markers were obtained: alpha fetoprotein, 71.8 ng/mL (normal range, up to 8.0 ng/mL); human chorionic gonadotropin (hCG), 2,003 mUI/mL (normal range, inferior to 5.0 mUI/mL), and lactate dehydrogenase, 546 UI/L (normal range, 120 to 246 UI/L).

The patient underwent a right inguinal orchiectomy on November 24, 2016, and the pathologic report was consistent with nonseminomatous germ cell tumor (NSGCT) in the form of embryonal carcinoma (immunohistochemistry: carcinoembryonic antigen, negative; hCG, negative; cancer antigen 125, negative; placental alkaline phosphatase, positive; C-KIT, negative; AE1 to AE3, positive; calretinin, negative; CD30, positive). Post-orchiectomy serum tumor markers were as follows: alpha fetoprotein, 159.4 ng/mL (normal range, up to 8.0 ng/mL); hCG, 2,661.3 mUI/mL (normal range, inferior to 5.0 mUI/mL); lactate dehydrogenase, 482 UI/L (normal range, 120 to 246 UI/L).

In the face of the findings of intermediate-risk NSGCT according to the International Germ Cell Cancer Collaborative Group classification, systemic therapy was proposed with bleomycin, etoposide, and cisplatin (BEP) for four cycles, which is the standard-of-care therapy in this setting.^1^ Considering the known G6PD deficiency, an extensive search of the literature was performed regarding the safety of chemotherapy drugs in this scenario, but almost no data were found. G6PD was dosed (33.1 mU per billion erythrocytes [normal, > 118 mU per billion erythrocytes]), and activity was consistent with moderate deficiency. A geneticist was consulted, and after considering risks and benefits, chemotherapy was started on December 1, 2016, with the patient on the oncology ward under rigorous daily surveillance.

**Fig 1:**
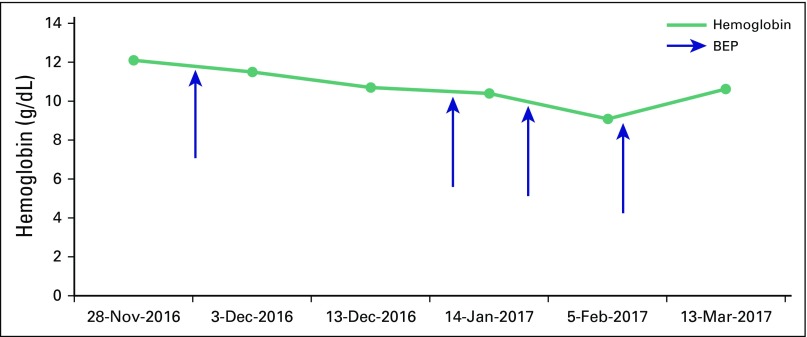
Hemoglobin trend. BEP, bleomycin, etoposide, and cisplatin.

**Fig 2:**
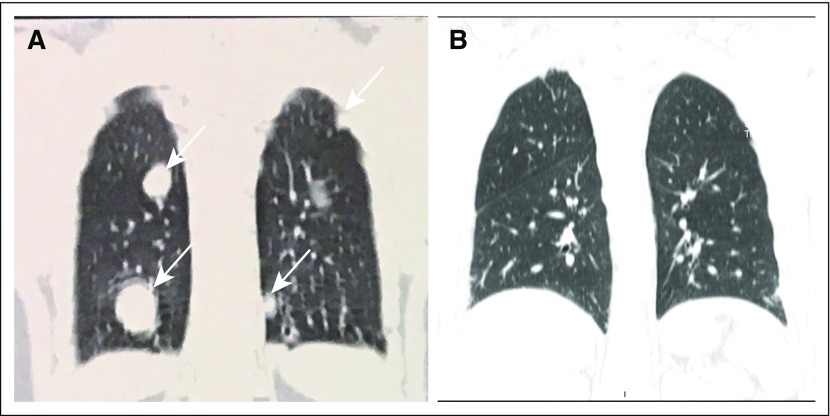
(A) Chest computed tomography on November 21, 2016, and (B) on March 13, 2017.

**Fig 3:**
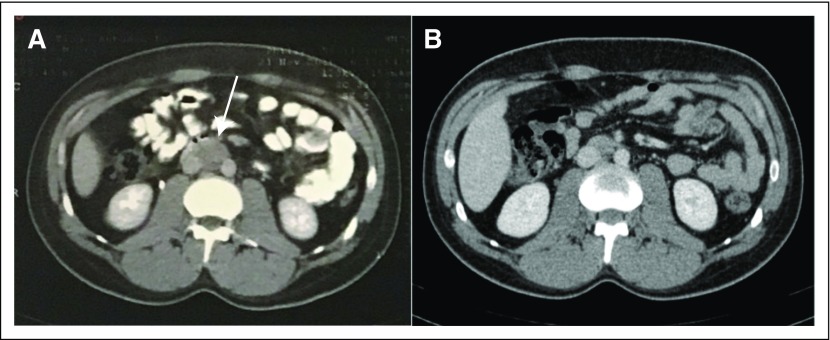
(A) Abdominal computed tomography on November 21, 2016, and (B) on March 13, 2017.

Despite the fear of acute hemolysis, laboratory analysis showed no remarkable variations of hemoglobin levels throughout the four cycles of BEP, as shown in Figure 1. The patient received standard doses of chemotherapy without any other special precaution except for adequate intravenous hydration. After the first cycle of BEP, he presented with deep venous thrombosis of a peripherally inserted central venous catheter in the right arm, but treatment was otherwise well tolerated. The patient had a complete response to chemotherapy, as seen by tumor markers (Table 1) and imaging studies (Figs 2A-2B and 3A-3B). The patient returned for follow-up in late March 2017 with no evidence of disease and will be observed regularly with serum tumor markers and computed tomography scans as per protocol for testicular germ cell tumors.

**Table 1:**
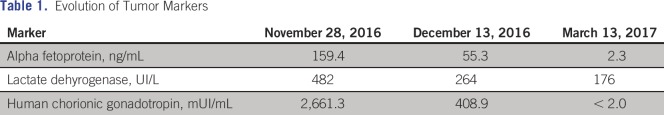
Evolution of Tumor Markers

## DISCUSSION

G6PD deficiency is an X-linked disorder that affects approximately 400 million people worldwide,^2,3^ being more frequent in regions where malaria is endemic such as in Asian and African countries.^4^ As an X-linked disease, it primarily affects boys. The diagnosis is made on the basis of direct measurements of G6PD activity in a population of red blood cells. Depending on the level of residual activity, it is classified as I or II (severe deficiency) or III (moderate deficiency); class IV and V have normal or higher levels of enzyme activity, so they have no clinical significance. The majority of affected patients have class II or class III deficiency and, although they are asymptomatic most of the time,^4^ exposure to oxidative stress situations can lead to acute hemolytic anemia. Crises are often manifested as cyanosis, headache, dyspnea, jaundice and, in severe cases, acute renal failure and even death.^3,5^

G6PD-deficient erythrocytes under oxidative stress have impaired production of nicotinamide adenine dinucleotide phosphate and thus are more susceptible to drug-induced lysis.^3,4^ Several drugs have been implicated in hemolysis in G6PD-deficient patients, but some authors have noted that certain antibacterials may have been mistakenly credited for hemolysis that in fact is attributable to the oxidative stress triggered by infection.^2,5^ Currently, besides antimalarial drugs, other drugs frequently related to hemolysis in this scenario are quinolones, nitrofurantoin, sulfadiazine, cotrimoxazole, phenazopyridine, toluidine blue, and rasburicase.^2,4,6^

In the recent years, multiple clinical reports^7-9^ describing severe adverse reactions associated with the use of rasburicase in patients with cancer who have a G6PD deficiency led regulatory agencies (US Food and Drug Administration, European Medicines Agency, and the Pharmaceuticals and Medical Devices Agency) to contraindicate its use in G6PD-deficient patients.^4^ Rasburicase is used for prophylaxis of hyperuricemia during chemotherapy in patients with tumors who are at high risk for tumor lysis syndrome (ie, lymphoma, leukemia, germ cell tumors, or small-cell lung cancer).^4^ A recombinant urate oxidase enzyme turns uric acid into more hydrophilic molecules (allantoin and hydrogen peroxide).^9^ The accumulation of hydrogen peroxide puts G6PD-deficient patients at risk for severe hemolytic anemia and possibly life-threatening methemoglobinemia.^4^

Except for these case reports of rasburicase, the description of chemotherapy use and its outcomes on G6PD-deficient patients are extremely limited in the literature.^3,10^ This may be related to the fact that most patients with cancer are never tested for G6PD deficiency or maybe because chemotherapy in these patients is uneventful, as in the patient described here. We also could not find specific recommendations on drug labels, which have obligated us to make decisions about chemotherapy solely by considering the potential benefit of treatment of germ cell tumors. We believe that more information is needed to support the safety of chemotherapy use for G6PD-deficient patients.
